# The ATRX cDNA is prone to bacterial IS10 element insertions that alter its structure

**DOI:** 10.1186/2193-1801-3-222

**Published:** 2014-05-02

**Authors:** David Valle-García, Lyra M Griffiths, Michael A Dyer, Emily Bernstein, Félix Recillas-Targa

**Affiliations:** Instituto de Fisiología Celular, Departamento de Genética Molecular, Universidad Nacional Autónoma de México, Ciudad Universitaria, México DF, México; Department of Oncological Sciences, Icahn School of Medicine at Mount Sinai, 1470 Madison Avenue, New York, NY 10029 USA; Department of Developmental Neurobiology, St. Jude Children’s Research Hospital, Memphis, TN USA; Howard Hughes Medical Institute, Chevy Chase, MD USA

**Keywords:** ATRX, Insertion element, Chromatin remodeling, Cloning vector, ATRX over-expression, IS10 element

## Abstract

The SWI/SNF-like chromatin-remodeling protein ATRX has emerged as a key factor in the regulation of α-globin gene expression, incorporation of histone variants into the chromatin template and, more recently, as a frequently mutated gene across a wide spectrum of cancers. Therefore, the availability of a functional ATRX cDNA for expression studies is a valuable tool for the scientific community. We have identified two independent transposon insertions of a bacterial IS10 element into exon 8 of *ATRX* isoform 2 coding sequence in two different plasmids derived from a single source. We demonstrate that these insertion events are common and there is an insertion hotspot within the ATRX cDNA. Such IS10 insertions produce a truncated form of ATRX, which significantly compromises its nuclear localization. In turn, we describe ways to prevent IS10 insertion during propagation and cloning of ATRX-containing vectors, including optimal growth conditions, bacterial strains, and suggested sequencing strategies. Finally, we have generated an insertion-free plasmid that is available to the community for expression studies of ATRX.

**Electronic supplementary material:**

The online version of this article (doi:10.1186/2193-1801-3-222) contains supplementary material, which is available to authorized users.

## Background

ATRX is an ATP-dependent chromatin-remodeling helicase of the SWI/SNF family and was originally identified because mutations in its coding region give rise to a complex disorder known as α-Thalassemia, Mental Retardation X-linked (ATRX) syndrome. Its role in the α-Thalassemia feature of this syndrome is through its regulation of the chromatin state of the α-globin genes (Higgs et al. 2005; Gibbons 2006; Law et al. 2010; Ratnakumar et al. 2012). However, its role in other ATRX syndrome traits remains largely unknown. Mechanistically, ATRX has been implicated in heterochromatin maintenance and formation, DNA methylation, regulation of gene expression, alternative lengthening of telomere suppression and histone variant deposition (for review see: Bérubé 2011; Ratnakumar and Bernstein 2013; Clynes et al. 2013). Recently, large-scale genome sequencing efforts have identified *ATRX* mutations and deletions in a range of tumor types, including glioblastoma multiforme, neuroblastoma and pancreatic neuroendocrine tumors (Jiao et al. 2011; Schwartzentruber et al. 2012; Cheung et al. 2012; Molenaar et al. 2012; Clynes et al. 2013). These alterations appear to result in loss of functional ATRX, suggesting that ATRX acts as tumor suppressor. However, the mechanisms by which such mutations result in ATRX loss-of-function remain unclear.

For the reasons noted above, ATRX has emerged as an important chromatin remodeler to study during normal development and disease. However, the cloning and overexpression of *ATRX* cDNA has been challenging due to its large size: 2,492 amino acids encoded in an 11,167 bp cDNA (isoform 1) and 2,454 amino acids encoded in an 11,031 bp cDNA (isoform 2). Here, we report two independent transposon insertions of an IS10 element into exon 8 of the *ATRX* isoform 2 coding sequence in two different plasmids derived from a single original source. Our data suggest that *ATRX* contains at least one insertion hot spot that is highly active when the plasmid is grown under standard bacterial conditions. Therefore, we recommend strategies to prevent IS10 insertions during propagation and cloning of *ATRX*-containing vectors. Furthermore, we analyzed the functional effect of this insertion and conclude that IS10 insertions produce a truncated form of ATRX that compromises its cellular distribution.

## Results and discussion

We sequenced two different ATRX plasmids that have been widely used for overexpression experiments (Additional file 1) (Bérubé et al. 2002,2008; McFarlane and Preston 2011; Ratnakumar et al. 2012; Newhart et al. 2012). One of the plasmids contains the full length *ATRX* cDNA sequence in a pEGFP (GFP-ATRX) (Bérubé et al. 2008) backbone and the other is cloned in a lentiviral backbone (ATRX-YFP) and was derived from the sequence contained in GFP-ATRX (Newhart et al. 2012). We found that the *ATRX* cDNA sequence that has been cloned corresponds to isoform 2, which lacks exon 6 in comparison to isoform 1. Exon 6 codes for 38 highly conserved amino acids. The functional relevance, if any, between isoform 1 and isoform 2 has yet to be reported. In addition, we found that E124 is missing in ATRX isoform 2. This amino acid is the last residue encoded by exon 5.

**Figure 1:**
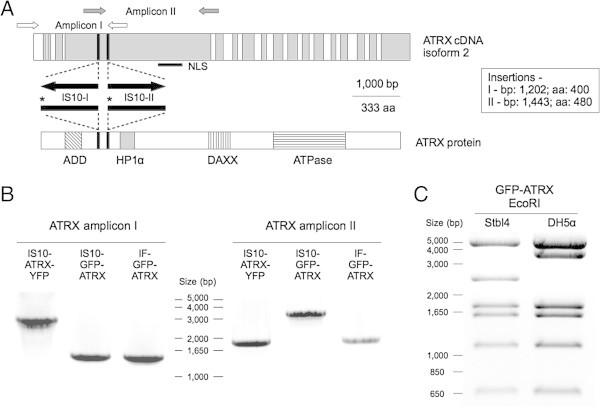
**ATRX overexpression plasmids contain IS10 insertions. (A)** Schematic map of the insertions found in IS10-GFP-ATRX and IS10-ATRX-YFP. The exon structure (alternate white and gray bars) of *ATRX* cDNA isoform 2 (top) and some relevant domains from the protein sequence (bottom) are shown. The insertion sites are denoted by black bars and their positions in the cDNA and the protein are described in the right box. Black arrows show the direction of the IS10 elements found. The stop codons introduced by IS10 in the protein sequence are indicated with asterisks. The position of two of the primer pairs used for analyzing ATRX sequence is shown (white and gray arrows). The NLS within *ATRX* cDNA is indicated with a black bar. **(B)** PCR analysis of IS10-GFP-ATRX, IS10-ATRX-YFP and IF-GFP-ATRX using the primers shown in (A). Both amplicons of IF-GFP-ATRX have the expected size (1,449 bp for amplicon I and 1,935 for amplicon II) while amplicon I shows an insertion in IS10-ATRX-YFP and amplicon II shows an insertion in IS10-GFP-ATRX (both have additional 1,336 bp). **(C)** Representative EcoRI digestion patterns of the IF-GFP-ATRX plasmid grown in two different bacteria strains. The Stbl4-derived plasmid has the expected pattern while the plasmid derived from DH5α shows an insertion in the 2,400bp fragment (shift from a 2,468 bp fragment to ~3,800 bp).

To our surprise, we uncovered transposon insertions of IS10 elements within the *ATRX* coding sequence in both plasmids analyzed (Figure 1A-B). The transposable prokaryotic *IS* family has been reported to be a common source of mutations in the *Escherichia coli* genome (Rodriguez et al. 1992). Likewise, it has also been shown that IS10-related sequences may lead to cloning aberrations and sequence contamination of plasmids hosted in *E. coli* (Rood et al. 1980; Müller et al. 1989; Kovarík et al. 2001; Kobori et al. 2009; Umenhoffer et al. 2010). Interestingly, the IS10 insertion in both plasmids - which we name IS10-GFP-ATRX and IS10-ATRX-YFP - was found in exon 8. However, sequencing and PCR analyses showed that both the insertion site and the direction of the IS10 element are different in each plasmid, suggesting independent insertion events (Figure 1A,B, Additional file 1). The insertion site within IS10-GFP-ATRX closely resembles the 9-bp canonical IS10 insertion sequence (Additional file 1; see Kovarík et al. , see Kovarík et al. see Kovarík et al. 2001), and may represent a hotspot for IS10 transposition. The insertion site within IS10-ATRX-YFP has a more divergent sequence. It is interesting to note that IS10-ATRX-YFP was derived from IS10-GFP-ATRX, however there is no sequence evidence of an IS10 excision in IS10-ATRX-YFP. This suggests that the *ATRX* cDNA of IS10-GFP-ATRX used to derive IS10-ATRX-YFP was lacking the IS10 insertion, and that the insertion observed in the latter was acquired during the cloning or plasmid expansion process. This highlights the dynamic feature of this transposable element.

In order to assess if the expression of the truncated form of ATRX derived from the IS10-GFP-ATRX plasmid may retain some of the characteristics of full length ATRX, we sub-cloned the *ATRX* cDNA sequence from the IS10-GFP-ATRX plasmid to generate an ‘Insertion-Free’ vector that we call IF-GFP-ATRX (see details in Methods). IF-GFP-ATRX was further analyzed by PCR and restriction analysis (data not shown) and the *ATRX* cDNA was fully sequenced to ensure that the insertion was completely removed (Additional file 1).

Next we investigated if the insertions are an isolated event or a common phenomenon. IF-GFP-ATRX was transformed into both DH5α and Stbl4 *E. coli* strains. The plasmids were analyzed by colony PCR with primers spanning the IS10 insertion sites found in IS10-ATRX-YFP and IS10-GFP-ATRX (Additional file 1, see Methods). We found that 18% of the DH5α colonies (26 out of 139 analyzed colonies) present evidence of insertions within exon 8. Sequencing and restriction analysis of the plasmid containing insertions revealed that the observed change is caused by *de novo* insertions of the IS10 transposable element (Figure 1C, Additional file 1). Conversely, the plasmid derived from the Stbl4 bacteria - a strain specifically designed for cloning unstable inserts - showed a significant lower insertion rate (3 out of 80 analyzed colonies – p-value < 0.01, binomial test). Furthermore, we found evidence of other aberrations in the DH5α colonies by restriction analyses (6 out of 30 analyzed colonies that did not contain the exon 8 IS10 insertion; data not shown). This suggests that the ATRX plasmid may be subjected to IS10 insertions in other regions of the *ATRX* cDNA or other types of alterations such as recombination events. This data strongly suggests that insertions within the *ATRX* coding sequence in *E. coli* are a common event that is influenced by the bacterial strain used to amplify ATRX-containing plasmids. Moreover, our results suggest that ATRX presents an IS10 insertion hotspot within exon 8. The causes that trigger the insertion of the IS10 element within the *ATRX* cDNA remain unclear. However, the homogeneity of the restriction patterns in the IF-GFP-ATRX with *de novo* IS10 insertions (Figure 1C) suggests that the IS10 transposition is an early clonal event. In fact, it has been proposed that the stress induced by the transformation process may be a trigger for IS10 transposition (Kovarík et al. 2001).

**Figure 2:**
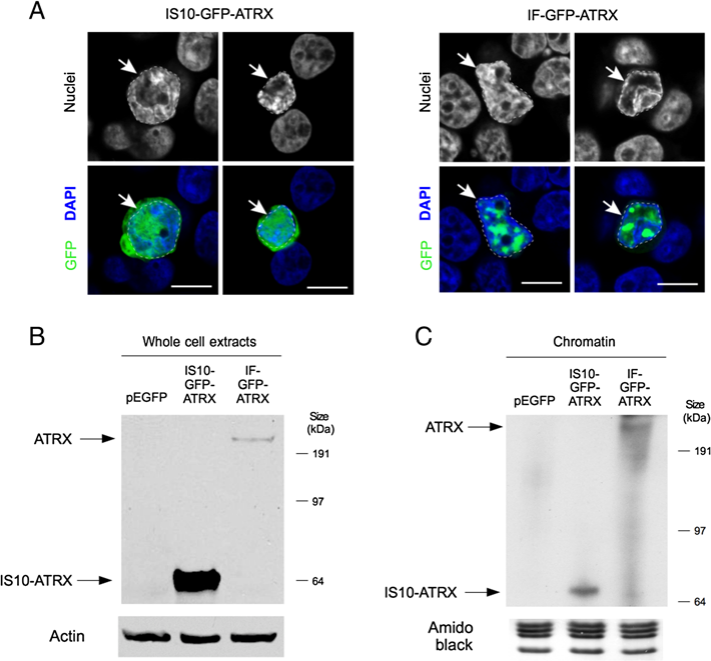
**IS10-element alters GFP-ATRX localization and incorporation into chromatin. (A)** HEK-293T cells were transiently transfected with IS10-GFP-ATRX (left) or IF-GFP-ATRX (right). The localization of GFP-ATRX was determined by confocal fluorescence microscopy 72 hours post-transfection. Representative images are shown. Dashed lines on the highlighted cells indicate nuclei area. IS10-GFP-ATRX displays a diffuse nuclear and cytoplasmic localization. Conversely, ATRX-GFP has a nuclear localization in the IF-GFP-ATRX transfected cells with a focal pattern. **(B)** Western analysis of whole cell protein extracts from the IS10-GFP-ATRX and IF-GFP-ATRX transfections against GFP (top) and actin (bottom). Bands corresponding to IF-GFP-ATRX and IS10-GFP-ATRX products are indicated with arrows. **(C)** Chromatin extracts from the IS10-GFP-ATRX and IF-GFP-ATRX transfections were analyzed with GFP antibodies (top). Bands corresponding to IF-GFP-ATRX and IS10-GFP-ATRX products are highlighted with arrows. Amido black staining of histones was used as a loading control (bottom). A truncated product is observed in the IS10-GFP-ATRX transfections.

We next transfected IS10-GFP-ATRX and IF-GFP-ATRX into HEK 293T cells and assessed ATRX expression and chromatin association. We found that the transfection efficiency was approximately double for IS10-GFP-ATRX (60%) compared to IF-GFP-ATRX (30%), and that the sub-cellular localization was dramatically different (data not shown, Figure 2A). As expected, IF-GFP-ATRX transfected cells show a focal nuclear localization of GFP-ATRX (Figure 2A) in agreement with previous reports (Xue et al. 2003; Tang et al. 2004; Ishov et al. 2004; Bérubé et al. 2008). However, we observed a clear cytoplasmic GFP signal in the IS10-GFP-ATRX transfected cells and a more diffuse nuclear localization (Figure 2A). According to our sequence analysis, a stop codon is introduced right after the IS10 insertion site in both plasmids containing the IS10 element (Figure 1A). As ATRX is N-terminally GFP-tagged, the fluorescence observed in the IS10-GFP-ATRX transfectants is likely due to the overexpression of a truncated GFP-ATRX fusion that contains only the ADD domain (Figure 1A).

Although we cannot exclude the possibility that the re-localization of the IS10-GFP-ATRX is due to an overexpression effect, ATRX nuclear localization is dependent on nuclear localization sequences (NLS) (Bérubé et al. 2008) and our sequence analysis predicts that the IS10-GFP-ATRX lacks the NLS (Figure1A). However, we note that although ATRX cytoplasmic localization is clear in the IS10-GFP-ATRX transfected cells, cells still do show some nuclear localization (Figure 2A). Because the ADD domain alone is capable of recognizing and binding the histone H3 tail *in vitro* by reading the combinatorial methylation state of histone H3 lysines 4 and 9 (Eustermann et al. 2011; Iwase et al. 2011) a small fraction of the expressed protein product may still be tethered to chromatin.

To probe this further and assay the chromatin association of the ATRX protein products, we prepared both whole cell and chromatin extracts from the transfected HEK 293T cells and performed Western blot analyses. As predicted, we found a truncated ATRX product from the transfections performed with the IS10-GFP-ATRX plasmid (Figure 2B,C). This truncated product is ~70 kDa and corresponds to the ATRX ADD domain coupled to the N-terminal GFP. This is further supported by the fact that the IS10-GFP-ATRX product is detectable only with a GFP antibody and not with an ATRX antibody whose epitope recognizes the C-terminal portion of the protein (data not shown).

While we observed chromatin-bound full-length ATRX from the IF-GFP-ATRX transfections, we noted that the IS10-GFP-ATRX truncated product also binds to the chromatin fraction (Figure 2C). This supports the idea that the ADD domain allows binding to chromatin. Whether the IS10-GFP-ATRX product has any effect on the chromatin landscape remains to be explored, for example, it could act as a dominant negative. However, it is interesting to note that in comparison to full-length ATRX, the IS10-GFP-ATRX fragment has a greater accumulation in whole cell extracts (Figure 2B) than in chromatin (Figure 2C). Taken together, these results strongly suggest that the IS10 element provokes the production of a truncated ATRX form that has a diffuse cellular localization, but still retains the capacity to bind to chromatin. Importantly, during experimental conditions, if one were to perform immunofluorescence of IS10-GFP-ATRX transduced cells without Western blot analysis, one might conclude that full length ATRX is expressed.

## Conclusions

Here we show that two ATRX overexpression plasmids, utilized in the ATRX research community, contain IS10-element insertions around a probable hot spot in exon 8 of *ATRX*. Further, we demonstrate that IS10 transposition events are common if *ATRX*-containing vectors are grown in standard bacteria strains. We note here that the dynamic nature of the insertions makes it likely that any single lab may have batches of contaminated and insertion-free plasmid. We further highlight that the published studies of ATRX function using these plasmids may have indeed been insertion-free, as we have confirmed for our own studies (Ratnakumar et al. 2012). However, because the IS10-element has a significant effect on ATRX sub-cellular localization and allows its incorporation into chromatin, experiments performed with IS10-ATRX-containing plasmids could generate inaccurate conclusions.

It has not escaped our attention that despite all the mutations identified in ATRX syndrome patients and, more recently, in distinct cancer types (Jiao et al. 2011; Schwartzentruber et al. 2012; Cheung et al. 2012; Clynes et al. 2013), few ATRX over-expression experiments mimicking such mutations have been published. It is our belief that to understand the contribution of ATRX mutations in disease etiology, we need to develop reliable over-expression systems. However, our results show that these experiments are challenging and subjected to technical hurdles that the community should be aware of.

Finally, we have made our IF-GFP-ATRX plasmid available through the AddGene database (plasmid 45444). Also, we provide technical advice for the propagation and cloning of ATRX-containing vectors (see Methods section). The sequences obtained from the IS10-GFP-ATRX and IS10-ATRX-YFP plasmids, as well as the primers used for the ATRX sequencing, cloning and analysis are included in the Additional file 1. Thus, we recommend that each laboratory should take the necessary steps to avoid IS10 contamination and test each plasmid preparation for IS10 insertion elements. We are hopeful that this technical resource will be useful for future studies and will help to avoid propagation and usage of IS10-ATRX-containing plasmids.

## Methods

### ATRX-containing plasmid propagation and handling

The ATRX plasmids were propagated using ElectroMAX™ Stbl4™ Competent Cells (Life Technologies, 11635-018). Top10 (Invitrogen, C404003) and DH5α bacteria were also used and are not recommended for growing *ATRX*-containing plasmids. We recommend growing bacteria at 30°C to avoid plasmid recombination. Using strains lacking IS-elements such as MDS42 is also recommended.

### ATRX PCR analysis and sequencing

PCR analyses were performed using Platinum® Pfx DNA Polymerase (Invitrogen 11708-013), according to manufacturer’s indications. The primers used span across the *ATRX* cDNA and their sequences can be found in Additional file 1. *ATRX* cDNA sequencing was performed using the same primers and one additional primer specific for exon 8 that can also be found in Additional file 1. We recommend analyzing the plasmid by PCR (or restriction digest) following culture in *E. coli* and on a frequent basis when working with *ATRX*-containing vectors.

### ATRX transformation and growth in different bacterial strains

ElectroMAX™ Stbl4™ competent cells (Life Technologies, 11635-018) and DH5α competent cells (Life Technologies 18258-012) were transformed according to manufacturer’s indications with 50 ng of IF-GFP-ATRX. Cells were plated and growth at 37°C. Colonies from three independent transfections were picked and diluted in 100μl of PBS. 5 μl were used for colony PCR using the ATRX IS10 primers from Additional file 1. Colonies that presented evidence of insertion, as well as control colonies were minipreped using the QIAprep Spin Miniprep Kit (Qiagen, 27106) 2 μg of the plasmid were digested with 2 units of EcoRI (New England Biolabs, R0101S) for 1 hour at 37°C.

### IF-GFP-ATRX construct generation

IS10-GFP-ATRX was digested with BamHI and AflII (NEB) to produce a 1200 bp band (Fragment A). A plasmid containing internal *ATRX* sequence was generated using GenScript services: (5′-ggtaccTACGTCAGCTTAAGGCTTTTAAGTCTGTGTTGGCTGATATTAAGAAGGCTCATCTTGCATTGGAAGAAGACTTAAATTCCGAGTTTCGAGCGATGGATGCTGTAAACAAAGAGAAAAATACCAAAGAGCATAAAGTCATAGATGCTAAGTTTGAAACAAAAGCACGAAAAGGAGAAAAACCTTGTGCTTTGGAAAAGAAGGATATTTCAAAGTCAGAAGCTAAACTTTCAAGAAAACAGGTAGATAGTGAGCACATGCATCAGAATGTTCCAACAGAGGAACAAAGAACAAATAAAAGTACCGGTGGTGAACATaagctt-3). A restriction digestion product was generated using the GenScript plasmid and enzymes, AflII and AgeI (Fragment B). A restriction digestion product of IS10-GFP-ATRX was generated using enzymes, AgeI (NEB) and XbaI (Fragment C). Finally, BamHI and XbaI (NEB) were used to digest pEGFP-C2 plasmid, generating a backbone for insertion of the *ATRX* fragments. The three *ATRX*-containing fragments (A, B, and C) were ligated first (1200 bp + 300 bp + 6000 bp). Then the backbone pEGFP vector was added for a second ligation reaction. Ligation product was transformed into dam^-^/dcm^-^ competent E. coli (NEB C2925H). Colonies were mini-prepped and sequenced.

### Transfections

Transfections were performed in the HEK-293T cell line (ATCC: CRL-11268) using Lipofectamine® 2000 transfection reagent according to manufacturer’s indications. 14 μg of each plasmid were transfected in 70% confluent 10 cm plates. Cells were harvested 48 hours post-transfection for chromatin preparation or fixed for microscopy (see below).

### Microscopy

24 hours post-transfection, cells were seeded onto chamber well slides. 48 hours later, cells were fixed with 4% paraformaldehyde/PBS (Sigma Aldrich) at 4°C overnight. Slides were stained with 4′,6-diamidino-2-phenylindole (DAPI) for 10 minutes at room temperature. Images were collected with a Zeiss LSM700 confocal microscope.

### Chromatin isolation and western analysis

Chromatin isolation was performed as described previously (Méndez and Stillman 2000) and chromatin samples were boiled at 100°C for 10 minutes instead of being sonicated. Western analysis was performed with the following antibodies: GFP (Santa Cruz sc-8334, 1:200 or Roche 1181460001, 1:10,000), Actin (Sigma-Aldrich A1978, 0.75 μg/mL). Histones from chromatin extracts were stained with amido black staining solution (Sigma A8181-1EA).

## Electronic supplementary material

Additional file 1: **Plasmid and primer sequences.** This files contains the sequences of the IS10 insertions, all the primers used for the analyses described on the paper, and the full IF-GP-ATRX sequence. (PDF 51 KB)

## Abbreviations

ATRX: α-Thalassemia, Mental Retardation X-linked gene.
