# Supplementary material for: The ATRX cDNA is prone to bacterial IS10 element insertions that alter its structure
Source: Springerplus. 2014 May 2;3:222. doi: 10.1186/2193-1801-3-222 (PMC4021028; doi:10.1186/2193-1801-3-222)
Supplement: Supplementary file 1 — Additional file 1: Plasmid and primer sequences. This files contains the sequences of the IS10 insertions, all the primers used for the analyses described on the paper, and the full IF-GP-ATRX sequence. (PDF 51 KB) [file 40064_2013_935_MOESM1_ESM.pdf]

>IS10-GFP-ATRX (exon 8 fragment, insertion sequence in orange)

AGTTGACAGTGAAAAGAGTAATAAAGTATATGAACATACATCCAGATTTTCTCCAAAGAAGACTAGTTCAAAT
TGTAATGGAGAAGAAAAGAAATTAGATGATTCCTGTTCTGGCTCTGTAACCTACTCTTATTCCGCACTAATTG
TGCCCAAAGAGATGATTAAGAAGGCAAAAAAACTGATTGAGACCACAGCCAACATGAACTCCAGTTATGTTAA
ATTTTTAAAGCAGGCAACAGATAATTCAGAAATCAGTTCTGCTACAAAATTACGTCAGCTTAAGGCTTTTAAG
TCTGTGTTGGCTGATATTAAGAAGGCTCATCTTGCATTGGAAGAAGACTTAAATTCCGAGTTTCGAGCGATGG
ATGCTGTAAACAAAGAGAAAAATACCAAAGAGCATAAAGTCATAGATGCTAAGTTCTGAGAGATCCCCTCATA
ATTTCCCCAAAGCGTAACCATGTGTGAATAAATTTTGAGCTAGTAGGGTTGCAGCCACGAGTAAGTCTTCCCT
TGTTATTGTGTAGCCAGAATGCCGCAAAACTTCCATGCCTAAGCGAACTGTTGAGAGTACGTTTCGATTTCTG
ACTGTGTTAGCCTGGAAGTGCTTGTCCCAACCTTGTTTCTGAGCATGAACGCCCGCAAGCCAACATGTTAGTT
GAAGCATCAGGGCGATTAGCAGCATGATATCAAAACGCTCTGAGCTGCTCGTTCGGCTATGGCGTAGGCCTAG
TCCGTAGGCAGGACTTTTCAAGTCTCGGAAGGTTTCTTCAATCTGCATTCGCTTCGAATAGATATTAACAAGT
TGTTTGGGTGTTCGAATTTCAACAGGTAAGTTAGTTGCTAGAACCCATGGCTCCTTTGCCGACGCTGAGTAGA
TTTTAGGTGACGGGTGGTGACAATGAGTCCGTGTCGAGCGCTGATTTTTTCGGCCTTTAGAGCGAGATTTATA
CAATAGAATTTGGCATGAGATTGGATTGCTTTTAGTCAGCCTCTTATAGCCTAAAGTCTTTGAGTGACTAGAT
GACATATCATGTAAGTTGCTGATANGTTTCCAGTTTTCCGCTCCTAGGTCTGCATATTGTACTTTTCCTCTTA
CTCGACTTAACCAGTACCAACCCAGCTTCTCAACGGATTTATACCATGGCACTTTAAAGCCAGCATCACTGAC
AATGAGCGGTGTGGTGTTACTCGGTAGAATGCTCGCAAGGTCGGCTAGAAATTGGTCATGAGCTTTCTTTGAA
CATTGCTCTGAAAGCGGGAACGCTTTCTCATAAAGAGTAACAGAACGACCGTGTAGTGCGACTGAAGCTCGCA
ATACCATAAGTCGTTTTTGCTCACGAATATCAGACCAGTCAACAAGTACAATGGGCATCGTATTGCCCGAACA
GATAAAGCTAGCATGCCAACGGTATACAGCGAGTCGCTCTTTGTGGAGGTGACGATTACCTAACAATCGGTCG
ATTCGTTTGATGTTATGTTTTGTTCTCGCTTTGGTTGGCAGGTTACGGCCAAGTTCGGTAAGAGTGAGAGTTT
TACAGTCAAGTAATGCGTGGCAAGCCAACGTTAAGCTGTTGAGTCGTTTTAAGTGTAATTCGGGGCAGAATTG
GTAAAGAGAGTCGTGTAAAATATCGAGTTCGCACATCTTGTTGTCTGATTATTGATTTTTCGCGAAACCATTT
GATCATATGACAAGATGTGTATCCACCTTAACTTAATGATTTTTACCAAAATCATTANGGGANTCATCAGTGC
TAAGTTTGAAACAAAAGCACGAAAAGGAGAAAAACCTTGTGCTTTGGAAAAGAAGGATATTTCAAAGTCAGAA
GCTAAACTTTCAAGAAAACAGGTAGATAGTGAGCACATGCATCAGAATGTTCCAACAGAGGAACAAAGAACAA
ATAAAAGTACCGGTGGTGAACATAAGAAATCTGATAGAAAAGAAGAACCTCAATATGAACCTGCCAACACTTC
TGAAGATTTAGACATGGATATTGTGTCTGTTCCTTCCTCAGTTCCAGAAGACATTTTTGAGAATCTTGAGACT
GCTATGGAAGTTCAGAGTTCAGTTGATCATCAAGGGGATGGCAGCAGTGGAACTGAACAAGAAGTGGAGAGTT
CATCTGTAAAATTAAATATTTCTTCAAAAGACAACAGAGGAGGTATTAAATCAAAAACTAC

>IS10-ATRX-YFP (exon 8 fragment, insertion sequence in blue)

ACAGATAATTCAGAAATCAGTTCTGCTACAAAATTACGTCAGCTTAAGGCTTTTAAGTCTGTGTTGGCTGATA
TTAAGAAGGCTCATCTTGCATTGGAAGAAGACTTAAATTCCGAGTTTCGAGCGATGGATGCTGTAAACAAAGA
GAAAAATACCAAAGAGCATAAAGTCATAGATGCTAAGTTTGAAACAAAAGCACGAAAAGGAGAAAAACCTTGT
GCTTTGGAAAAGAAGGATATTTCAAAGTCAGAAGCTAAACTTTCAAGAAAACAGGTAGATAGTGAGCACATGC
ATCAGAATGTTCCAACAGAGGAACAAAGAACAAATAAAAGTACCGGTGGTGAACATAAGAAATCTGATAGAAA
AGAAGAACCTCAATATGAACCTGCCAACACTTCTGAAGATTTAGACATGGATATTGTGTCCTGATGAATCCCC
TAATGATTTTTATCAAAATCATTAAGTTAAGGTAGATACACATCTTGTCATATGATCAAATGGTTTCGCCAAA
AATCAATAATCAGACAACAAAATGTGCGAACTCTATATTTTACACGACTCTCTTTACCAATTCTGCCCCGAAT
TACACTTAAAACGACTCAACAGCTTAACGTTGGCTTGCCACGCCTTACTTGACTGTAAAACTCTCACTCTTAC
CGAACTTGGCCGTAACCTGCCAACCAAAGCGAGAACAAAACATAACATCAAACGAATCGACCGATTGTTAGGT
AATCGTCACCTCCACAAAGAGCGACTCGCTGTATACCGTTGGCATGCTAGCTTTATCTGTTCGGGCAATACGA
TGCCCATTGTACTTGTTGACTGGTCTGATATCCGTGAGCAAAAACGGCTTATGGTATTGCGAGCTTCAGTCGC
ACTACACGGTCGTTCTGTTACTCTTTATGAGAAAGCGTTCCCGCTTTCAGAGCAATGTTCAAAGAAAGCTCAT
GACCAATTTCTAGCCGACCTTGCGAGCATTCTACCGAGTAACACCACACCGCTCATTGTCAGTGATGCTGGCT
TTAAAGTGCCATGGTATAAATCCGTTGAGAAGCTGGGTTGGTACTGGTTAAGTCGAGTAAGAGGACAAGTACA
ATATGCAGACCTAGGAGCGGAAAACTGGAAACCTATCAGCAACTTACATGATATGTCATCTAGTCACTCAAAG
ACTTTAGGCTATAAGAGGCTGACTAAAAGCAATCCAATCTCATGCCAAATTCTATTGTATAAATCTCGCTCTA
AAGGCCGAAAAAATCAGCGCTCGACACGGACTCATTGTCACCACCCGTCACCTAAAATCTACTCAGCGTCGGC
AAAGGAGCCATGGATTCTAGCAACTAACTTACCTGTTGAAATTCGAACACCCAAACAACTTGTTAATATCTAT
TCGAAGCGAATGCAGATTGAAGAAACCTTCCGAGACTTGAAAAGTCCTGCCTACGGACTAGGCCTACGCCATA
GCCGAACGAGCAGCTCAGAGCGTTTTGATATCATGCTGCTAATCGCCCTGATGCTTCAACTAACATGTTGGCT
TGCGGGCGTTCATGCTCAGAAACAAGGTTGGGACAAGCACTTCCAGGCTAACACAGTCAGAAATCGAAACGTA
CTCTCAACAGTTCGCTTAGGCATGGAAGTTTTGCGGCATTCTGGCTACACAATAACAAGGGAAGACTTACTCG

TGGCTGCAACCCTACTAGCTCAAAATTTATTCACACATGGTTACGCTTTGGGGAAATTATGAGGGGATCTCTC
AGTATTGTGTCTGTTCCTTCCTCAGTTCCAGAAGACATTTTTGAGAATCTTGAGACTGCTATGGAAGTTCAGA
GTTCAGTTGATCATCAAGGGGATGGCAGCAGTGGAACTGAACAAGAAGTGGAGAGTTCATCTGTAAAATTAAA
TATTTCTTCAAAAGACAACAGAGGAGGTATTAAATCAAAAACTACAGCTAAAGTAACAAAAGAATTATATGTT
AAACTCACTCCTGTTTCCCTTTCTAATTCCCCAATTAAAGGTGCTGATTGTCAGGAAGTTCCACAAGATAAAG
ATGGCTATAAAAGTTGTGGT

>IS10 de novo insertion in IF-GFP-ATRX grown in DH5α bacteria (exon 8 fragment, insertion sequence in green)

GGAGTACAGTTGAAGTGAAAGATAATAAAGTATATGAACATACATCCAGATTTTCTCCAAGAAGACTAGTTCA
AATTGTAATGGAGAAGAAAAGAAATTAGATGATTCCTGTTCTGGCTCTGTAACCTACTCTTATTCCGCACTAA
TTGTGCCCAAAGAGATGATTAAGAAGGCAAAAAAACTGATTGAGACCACAGCCAACATGAACTCCAGTTATGT
TAAATTTTTAAAGCAGGCAACAGATAATTCAGAAATCAGTTCTGCTACAAAATTACGTCAGCTTAAGGCTTTT
AAGTCTGTGTTGGCTGATATTAAGAAGGCTCATCTTGCATTGGAAGAAGACTTAAATTCCGAGTTTCGAGCGA
TGGATGCTGTAAACAAAGAGAAAAATACCAAAGAGCATAAAGTCATAGATGCTAAGTTCTGATGAATCCCCTA
ATGATTTTGGTAAAAATCATTAAGTTAAGGTGGATACACATCTTGTCATATGATCAAATGGTTTCGCGAAAAA
TCAATAATCAGACAACAAGATGTGCGAACTCGATATTTTACACGACTCTCTTTACCAATTCTGCCCCGAATTA
CACTTAAAACGACTCAACAGCTTAACGTTGGCTTGCCACGCATTACTTGACTGTAAAACTCTCACTCTTACCG
AACTTGGCCGTAACCTGCCAACCAAAGCGAGAACAAAACATAACATCAAACGAATCGACCGATTGTTAGGTAA
TCGTCACCTCCACAAAGAGCGACTCGCTGTATACCGTTGGCATGCTAGCTTTATCTGTTCGGGCAATACGATG
CCCATTGTACTTGTTGACTGGTCTGATATTCGTGAGCAAAAACGACTTATGGTATTGCGAGCTTCAGTCGCAC
TACACGGTCGTTCTGTTACTCTTTATGAGAAAGCGTTCCCGCTTTCAGAGCAATGTTCAAAGAAAGCTCATGA
CCAATTTCTAGCCGACCTTGCGAGCATTCTACCGAGTAACACCACACCGCTCATTGTCAGTGATGCTGCTTTA
AAGTGCCATGTATAAATCCGTTGAGAAGCTGGGTTGGTACTGGTTAAGTCGAGTAAGAGGACAAGTACAATAT
GCAGACCTAGGAGCGGAAAACTGGAAACCTATCAGCAACTTACATGATATGTCATCTAGTCACTCAAAGACTT
TAGGCTATAAGAGGCTGACTAAAAGCAATCCAATCTCATGCCAAATTCTATTGTATAAATCTCGCTCTAAAGG
CCGAAAAAATCAGCGCTCGACACGGACTCATTGTCACCACCCGTCACCTAAAATCTACTCAGCGTCGGCAAAG
GAGCCATGGATTCTAGCAACTAACTTACCTGTTGAAATTCGAACACCCAAACAACTTGTTAATATCTATTCGA
AGCGAATGCAGGTTGAAGAAACCTTCCGAGACTTGAAAAGTCCTGCCTACGGACTAGGCCTACGCCATAGCCG
AACGAGCAGCTCAGAGCGTTTTGATATCATGCTGCTAATCGCCCTGATGCTTCAACTAACATGTTGGCTTGCG
GGCGTTCATGCTCAGAAACAAGGATGGGACAAGCACTTCCAGGCTAACACAGTCAGAAATCGAAACGTACTCT
CAACAGTTCGCTTAGGCATGGAAGTTTTGCGGCATTCTGGCTACACAATAACAAGGGAAGACTTACTCGTGGC
TGCAACCCTACTAGCTCAAAATTTATTCACACATGGTTACGCTTTGGGGAAATTATGAGGGGATCTCTCAGTT
TGAAACAAAAGCACGAAAAGGAGAAAAACCTTGTGCTTTGGAAAAGAAGGATATTTCAAAGTCAGAAGCTAAA
CTTTCAAGAAAACAGGTAGATAGTGAGCACATGCATCAGAATGTTCCAACAGAGGAACAAAGAACAAATAAAA
GTACCGG

>ATRX exon 8 sequence showing insertion sites in IS10-GFP-ATRX / IF-GFP-ATRX (red) and IS10-ATRX-YFP (blue) plasmids

GTGGTGTGCGGAAGGTGGAAACTTGATTTGTTGTGACTTTTGCCATAATGCTTTCTGCAAGAAATGCATTCTA
CGCAACCTTGGTCGAAAGGAGTTGTCCACAATAATGGATGAAAACAACCAATGGTATTGCTACATTTGTCACC
CAGAGCCTTTGTTGGACTTGGTCACTGCATGTAACAGCGTATTTGAGAATTTAGAACAGTTGTTGCAGCAAAA
TAAGAAGAAGATAAAAGTTGACAGTGAAAAGAGTAATAAAGTATATGAACATACATCCAGATTTTCTCCAAAG
AAGACTAGTTCAAATTGTAATGGAGAAGAAAAGAAATTAGATGATTCCTGTTCTGGCTCTGTAACCTACTCTT
ATTCCGCACTAATTGTGCCCAAAGAGATGATTAAGAAGGCAAAAAAACTGATTGAGACCACAGCCAACATGAA
CTCCAGTTATGTTAAATTTTTAAAGCAGGCAACAGATAATTCAGAAATCAGTTCTGCTACAAAATTACGTCAG
CTTAAGGCTTTTAAGTCTGTGTTGGCTGATATTAAGAAGGCTCATCTTGCATTGGAAGAAGACTTAAATTCCG
AGTTTCGAGCGATGGATGCTGTAAACAAAGAGAAAAATACCAAAGAGCATAAAGTCATAGA**TGCTAAGTT**TGA
AACAAAAGCACGAAAAGGAGAAAAACCTTGTGCTTTGGAAAAGAAGGATATTTCAAAGTCAGAAGCTAAACTT
TCAAGAAAACAGGTAGATAGTGAGCACATGCATCAGAATGTTCCAACAGAGGAACAAAGAACAAATAAAAGTA
CCGGTGGTGAACATAAGAAATCTGATAGAAAAGAAGAACCTCAATATGAACCTGCCAACACTTCTGAAGATTT
AGACATGGA**TATTGTGTC**TGTTCCTTCCTCAGTTCCAGAAGACATTTTTGAGAATCTTGAGACTGCTATGGAA
GTTCAGAGTTCAGTTGATCATCAAGGGGATGGCAGCAGTGGAACTGAACAAGAAGTGGAGAGTTCATCTGTAA
AATTAAATATTTCTTCAAAAGACAACAGAGGAGGTATTAAATCAAAAACTACAGCTAAAGTAACAAAAGAATT
ATATGTTAAACTCACTCCTGTTTCCCTTTCTAATTCCCCAATTAAAGGTGCTGATTGTCAGGAAGTTCCACAA
GATAAAGATGGCTATAAAAGTTGTGGTCTGAACCCCAAGTTAGAGAAATGTGGACTTGGACAGGAAAACAGTG

ATAATGAGCATTTGGTTGAAAATGAAGTTTCATTACTTTTAGAGGAATCTGATCTTCGAAGATCCCCACGTGT
AAAGACTACACCCTTGAGGCGACCGACAGAAACTAACCCTGTAACATCTAATTCAGATGAAGAATGTAATGAA
ACAGTTAAGGAGAAACAAAAACTATCAGTTCCAGTGAGAAAAAAGGATAAGCGTAATTCTTCTGACAGTGCTA
TAGATAATCCTAAGCCTAATAAATTGCCAAAATCTAAGCAATCAGAGACTGTGGATCAAAATTCAGATTCTGA
TGAAATGCTAGCAATCCTCAAAGAGGTGAGCAGGATGAGTCACAGTTCTTCTTCAGATACTGATATTAATGAA
ATTCATACAAACCATAAGACTTTGTATGATTTAAAGACTCAGGCGGGGAAAGATGATAAAGGAAAAAGGAAAC
GAAAAAGTTCTACATCTGGCTCAGATTTTGATACTAAAAAGGGCAAATCAGCTAAGAGCTCTATAATTTCTAA
AAAGAAACGACAAACCCAGTCTGAGTCTTCTAATTATGACTCAGAATTAGAAAAAGAGATAAAGAGCATGAGT
AAAATTGGTGCTGCCAGAACCACCAAAAAAAGAATTCCAAATACAAAAGATTTTGACTCTTCTGAAGATGAGA
AACACAGCAAAAAAGGAATGGATAATCAAGGGCACAAAAATTTGAAGACCTCACAAGAAGGATCATCTGATGA
TGCTGAAAGAAAACAAGAGAGAGAGACTTTCTCTTCAGCAGAAGGCACAGTTGATAAAGACACGACCATCATG
GAATTAAGAGATCGACTTCCTAAGAAGCAGCAAGCAAGTGCTTCCACTGATGGTGTCGATAAGCTTTCTGGGA
AAGAGCAGAGTTTTACTTCTTTGGAAGTTAGAAAAGTTGCTGAAACTAAAGAAAAGAGCAAGCATCTCAAAAC
CAAAACATGTAAAAAAGTACAGGATGGCTTATCTGATATTGCAGAGAAATTCCTAAAGAAAGACCAGAGCGAT
GAAACTTCTGAAGATGATAAAAAGCAGAGCAAAAAGGGAACTGAAGAAAAAAAGAAACCTTCAGACTTTAAGA
AAAAAGTAATTAAAATGGAACAA

>IF-GFP-ATRX (backbone pEGFP-C2; green - GFP; purple – ATRX cDNA isoform 2; green background – start codon; red background – stop codon; pink - HA tag; gray – codons)

TAGTTATTAATAGTAATCAATTACGGGGTCATTAGTTCATAGCCCATATATGGAGTTCCGCGTTACATAACTT
ACGGTAAATGGCCCGCCTGGCTGACCGCCCAACGACCCCCGCCCATTGACGTCAATAATGACGTATGTTCCCA
TAGTAACGCCAATAGGGACTTTCCATTGACGTCAATGGGTGGAGTATTTACGGTAAACTGCCCACTTGGCAGT
ACATCAAGTGTATCATATGCCAAGTACGCCCCCTATTGACGTCAATGACGGTAAATGGCCCGCCTGGCATTAT
GCCCAGTACATGACCTTATGGGACTTTCCTACTTGGCAGTACATCTACGTATTAGTCATCGCTATTACCATGG
TGATGCGGTTTTGGCAGTACATCAATGGGCGTGGATAGCGGTTTGACTCACGGGGATTTCCAAGTCTCCACCC
CATTGACGTCAATGGGAGTTTGTTTTGGCACCAAAATCAACGGGACTTTCCAAAATGTCGTAACAACTCCGCC
CCATTGACGCAAATGGGCGGTAGGCGTGTACGGTGGGAGGTCTATATAAGCAGAGCTGGTTTAGTGAACCGTC
AGATCCGCTAGCGCTACCGGTCGCCACC**ATG**GTGAGCAAGGGCGAGGAGCTGTTCACCGGGGTGGTGCCCATC
CTGGTCGAGCTGGACGGCGACGTAAACGGCCACAAGTTCAGCGTGTCCGGCGAGGGCGAGGGCGATGCCACCT
ACGGCAAGCTGACCCTGAAGTTCATCTGCACCACCGGCAAGCTGCCCGTGCCCTGGCCCACCCTCGTGACCAC
CCTGACCTACGGCGTGCAGTGCTTCAGCCGCTACCCCGACCACATGAAGCAGCACGACTTCTTCAAGTCCGCC
ATGCCCGAAGGCTACGTCCAGGAGCGCACCATCTTCTTCAAGGACGACGGCAACTACAAGACCCGCGCCGAGG
TGAAGTTCGAGGGCGACACCCTGGTGAACCGCATCGAGCTGAAGGGCATCGACTTCAAGGAGGACGGCAACAT
CCTGGGGCACAAGCTGGAGTACAACTACAACAGCCACAACGTCTATATCATGGCCGACAAGCAGAAGAACGGC
ATCAAGGTGAACTTCAAGATCCGCCACAACATCGAGGACGGCAGCGTGCAGCTCGCCGACCACTACCAGCAGA
ACACCCCCATCGGCGACGGCCCCGTGCTGCTGCCCGACAACCACTACCTGAGCACCCAGTCCGCCCTGAGCAA
AGACCCCAACGAGAAGCGCGATCACATGGTCCTGCTGGAGTTCGTGACCGCCGCCGGGATCACTCTCGGCATG
GACGAGCTGTACAAGTCCGGCCGGACTCAGATCTCGAGGTCGACGGTATCGATAAGCTTGATATCGAATTCCT
GCAGCCCGGGGGATCCGCCCCAGCAATCACAGAAGCCGACAAGGCGTTCAAGCGAAAACATGACCGCTGAGCC
CATGAGTGAAAGCAAGTTGAATACATTGGTGCAGAAGCTTCATGACTTCCTTGCACACTCATCAGAAGAATCT
GAAGAAACAAGTTCTCCTCCACGACTTGCAATGAATCAAAACACAGATAAAATCAGTGGTTCTGGAAGTAACT
CTGATATGATGGAAAACAGCAAGGAAGAGGGAACTAGCTCTTCAGAAAAATCCAAGTCTTCAGGATCGTCACG
ATCAAAGAGGAAACCTTCAATTGTAACAAAGTATGTAGAATCAGATGATGAAAAACCTTTGGATGATGAAACT
GTAAATGAAGATGCGTCTAATGAAAATTCAGAAAATGATATTACTATGCAGAGCTTGCCAAAAGGTACAGTGA
TTGTACAGCCAGAGCCAGTGCTGAATGAAGACAAAGATGATTTTAAAGGGCCTGAATTTAGAAGCAGAAGTAA
AATGAAAACTGAAAATCTCAAAAAACGCGGAGAAGATGGGCTTCATGGGATTGTGAGCTGCACTGCTTGTGGA
CAACAGGTCAATCATTTTCAAAAAGATTCCATTTATAGACACCCTTCATTGCAAGTTCTTATTTGTAAGAATT
GCTTTAAGTATTACATGAGTGATGATATTAGCCGTGACTCAGATGGAATGGATGAACAATGTAGGTGGTGTGC
GGAAGGTGGAAACTTGATTTGTTGTGACTTTTGCCATAATGCTTTCTGCAAGAAATGCATTCTACGCAACCTT
GGTCGAAAGGAGTTGTCCACAATAATGGATGAAAACAACCAATGGTATTGCTACATTTGTCACCCAGAGCCTT
TGTTGGACTTGGTCACTGCATGTAACAGCGTATTTGAGAATTTAGAACAGTTGTTGCAGCAAAATAAGAAGAA
GATAAAAGTTGACAGTGAAAAGAGTAATAAAGTATATGAACATACATCCAGATTTTCTCCAAAGAAGACTAGT
TCAAATTGTAATGGAGAAGAAAAGAAATTAGATGATTCCTGTTCTGGCTCTGTAACCTACTCTTATTCCGCAC
TAATTGTGCCCAAAGAGATGATTAAGAAGGCAAAAAAACTGATTGAGACCACAGCCAACATGAACTCCAGTTA
TGTTAAATTTTTAAAGCAGGCAACAGATAATTCAGAAATCAGTTCTGCTACAAAATTACGTCAGCTTAAGGCT
TTTAAGTCTGTGTTGGCTGATATTAAGAAGGCTCATCTTGCATTGGAAGAAGACTTAAATTCCGAGTTTCGAG

CGATGGATGCTGTAAACAAAGAGAAAAATACCAAAGAGCATAAAGTCATAGATGCTAAGTTTGAAACAAAAGC
ACGAAAAGGAGAAAAACCTTGTGCTTTGGAAAAGAAGGATATTTCAAAGTCAGAAGCTAAACTTTCAAGAAAA
CAGGTAGATAGTGAGCACATGCATCAGAATGTTCCAACAGAGGAACAAAGAACAAATAAAAGTACCGGTGGTG
AACATAAGAAATCTGATAGAAAAGAAGAACCTCAATATGAACCTGCCAACACTTCTGAAGATTTAGACATGGA
TATTGTGTCTGTTCCTTCCTCAGTTCCAGAAGACATTTTTGAGAATCTTGAGACTGCTATGGAAGTTCAGAGT
TCAGTTGATCATCAAGGGGATGGCAGCAGTGGAACTGAACAAGAAGTGGAGAGTTCATCTGTAAAATTAAATA
TTTCTTCAAAAGACAACAGAGGAGGTATTAAATCAAAAACTACAGCTAAAGTAACAAAAGAATTATATGTTAA
ACTCACTCCTGTTTCCCTTTCTAATTCCCCAATTAAAGGTGCTGATTGTCAGGAAGTTCCACAAGATAAAGAT
GGCTATAAAAGTTGTGGTCTGAACCCCAAGTTAGAGAAATGTGGACTTGGACAGGAAAACAGTGATAATGAGC
ATTTGGTTGAAAATGAAGTTTCATTACTTTTAGAGGAATCTGATCTTCGAAGATCCCCACGTGTAAAGACTAC
ACCCTTGAGGCGACCGACAGAAACTAACCCTGTAACATCTAATTCAGATGAAGAATGTAATGAAACAGTTAAG
GAGAAACAAAAACTATCAGTTCCAGTGAGAAAAAAGGATAAGCGTAATTCTTCTGACAGTGCTATAGATAATC
CTAAGCCTAATAAATTGCCAAAATCTAAGCAATCAGAGACTGTGGATCAAAATTCAGATTCTGATGAAATGCT
AGCAATCCTCAAAGAGGTGAGCAGGATGAGTCACAGTTCTTCTTCAGATACTGATATTAATGAAATTCATACA
AACCATAAGACTTTGTATGATTTAAAGACTCAGGCGGGGAAAGATGATAAAGGAAAAAGGAAACGAAAAAGTT
CTACATCTGGCTCAGATTTTGATACTAAAAAGGGCAAATCAGCTAAGAGCTCTATAATTTCTAAAAAGAAACG
ACAAACCCAGTCTGAGTCTTCTAATTATGACTCAGAATTAGAAAAAGAGATAAAGAGCATGAGTAAAATTGGT
GCTGCCAGAACCACCAAAAAAAGAATTCCAAATACAAAAGATTTTGACTCTTCTGAAGATGAGAAACACAGCA
AAAAAGGAATGGATAATCAAGGGCACAAAAATTTGAAGACCTCACAAGAAGGATCATCTGATGATGCTGAAAG
AAAACAAGAGAGAGAGACTTTCTCTTCAGCAGAAGGCACAGTTGATAAAGACACGACCATCATGGAATTAAGA
GATCGACTTCCTAAGAAGCAGCAAGCAAGTGCTTCCACTGATGGTGTCGATAAGCTTTCTGGGAAAGAGCAGA
GTTTTACTTCTTTGGAAGTTAGAAAAGTTGCTGAAACTAAAGAAAAGAGCAAGCATCTCAAAACCAAAACATG
TAAAAAAGTACAGGATGGCTTATCTGATATTGCAGAGAAATTCCTAAAGAAAGACCAGAGCGATGAAACTTCT
GAAGATGATAAAAAGCAGAGCAAAAAGGGAACTGAAGAAAAAAAGAAACCTTCAGACTTTAAGAAAAAAGTAA
TTAAAATGGAACAACAGTATGAATCTTCATCTGATGGCACTGAAAAGTTACCTGAGCGAGAAGAAATTTGTCA
TTTTCCTAAGGGCATAAAACAAATTAAGAATGGAACAACTGATGGAGAAAAGAAAAGTAAAAAAATAAGAGAT
AAAACTTCTAAAAAGAAGGATGAATTATCTGATTATGCTGAGAAGTCAACAGGGAAAGGAGATAGTTGTGACT
CTTCAGAGGATAAAAAGAGTAAGAATGGAGCATATGGTAGAGAGAAGAAAAGGTGCAAGTTGCTTGGAAAGAG
TTCAAGGAAGAGACAAGATTGTTCATCATCTGATACTGAGAAATATTCCATGAAAGAAGATGGTTGTAACTCT
TCTGATAAGAGACTGAAAAGAATAGAATTGAGGGAAAGAAGAAATTTAAGTTCAAAGAGAAATACTAAGGAAA
TACAAAGTGGCTCATCATCATCTGATGCTGAGGAAAGTTCTGAAGATAATAAAAAGAAGAAGCAAAGAACTTC
ATCTAAAAAGAAGGCAGTCATTGTCAAGGAGAAAAAGAGAAACTCCCTAAGAACAAGCACTAAAAGGAAGCAA
GCTGACATTACATCCTCATCTTCTTCTGATATAGAAGATGATGATCAGAATTCTATAGGTGAGGGAAGCAGCG
ATGAACAGAAAATTAAGCCTGTGACTGAAAATTTAGTGCTGTCTTCACATACTGGATTTTGCCAATCTTCAGG
AGATGAAGCCTTATCTAAATCAGTGCCTGTCACAGTGGATGATGATGATGACGACAATGATCCTGAGAATAGA
ATTGCCAAGAAGATGCTTTTAGAAGAAATTAAAGCCAATCTTTCCTCTGATGAGGATGGATCTTCAGATGATG
AGCCAGAAGAAGGGAAAAAAAGAACTGGAAAACAAAATGAAGAAAACCCAGGAGATGAGGAAGCAAAAAATCA
AGTCAATTCTGAATCAGATTCAGATTCTGAAGAATCTAAGAAGCCAAGATACAGACATAGGCTTTTGCGGCAC
AAATTGACTGTGAGTGACGGAGAATCTGGAGAAGAAAAAAAGACAAAGCCTAAAGAGCATAAAGAAGTCAAAG
GCAGAAACAGAAGAAAGGTGAGCAGTGAAGATTCAGAAGATTCTGATTTTCAGGAATCAGGAGTTAGTGAAGA
AGTTAGTGAATCCGAAGATGAACAGCGGCCCAGAACAAGGTCTGCAAAGAAAGCAGAGTTGGAAGAAAATCAG
CGGAGCTATAAACAGAAAAAGAAAAGGCGACGTATTAAGGTTCAAGAAGATTCATCCAGTGAAAACAAGAGTA
ATTCTGAGGAAGAAGAGGAGGAAAAAGAAGAGGAGGAGGAAGAGGAGGAGGAGGAGGAAGAGGAGGAGGAAGA
TGAAAATGATGATTCCAAGTCTCCTGGAAAAGGCAGAAAGAAAATTCGGAAGATTCTTAAAGATGATAAACTG
AGAACAGAAACACAAAATGCTCTTAAGGAAGAGGAAGAGAGACGAAAACGTATTGCTGAGAGGGAGCGTGAGC
GAGAAAAATTGAGAGAGGTGATAGAAATTGAAGATGCTTCACCCACCAAGTGTCCAATAACAACCAAGTTGGT
TTTAGATGAAGATGAAGAAACCAAAGAACCTTTAGTGCAGGTTCATAGAAATATGGTTATCAAATTGAAACCC
CATCAAGTAGATGGTGTTCAGTTTATGTGGGATTGCTGCTGTGAGTCTGTGAAAAAAACAAAGAAATCTCCAG
GTTCAGGATGCATTCTTGCCCACTGTATGGGCCTTGGTAAGACTTTACAGGTGGTAAGTTTTCTTCATACAGT
TCTTTTGTGTGACAAACTGGATTTCAGCACGGCGTTAGTGGTTTGTCCTCTTAATACTGCTTTGAATTGGATG
AATGAATTTGAGAAGTGGCAAGAGGGATTAAAAGATGATGAGAAGCTTGAGGTTTCTGAATTAGCAACTGTGA
AACGTCCTCAGGAGAGAAGCTACATGCTGCAGAGGTGGCAAGAAGATGGTGGTGTTATGATCATAGGCTATGA
GATGTATAGAAATCTTGCTCAAGGAAGGAATGTGAAGAGTCGGAAACTTAAAGAAATATTTAACAAAGCTTTG
GTTGATCCAGGCCCTGATTTTGTTGTTTGTGATGAAGGCCATATTCTAAAAAATGAAGCATCTGCTGTTTCTA
AAGCTATGAATTCTATACGATCAAGGAGGAGGATTATTTTAACAGGAACACCACTTCAAAATAACCTAATTGA
GTATCATTGTATGGTTAATTTTATCAAGGAAAATTTACTTGGATCCATTAAGGAGTTCAGGAATAGATTTATA
AATCCAATTCAAAATGGTCAGTGTGCAGATTCTACCATGGTAGATGTCAGAGTGATGAAAAAACGTGCTCACA
TTCTCTATGAGATGTTAGCTGGATGTGTTCAGAGGAAAGATTATACAGCATTAACAAAATTCTTGCCTCCAAA

ACACGAATATGTGTTAGCTGTGAGAATGACTTCTATTCAGTGCAAGCTCTATCAGTACTACTTAGATCACTTA
ACAGGTGTGGGCAATAATAGTGAAGGTGGAAGAGGAAAGGCAGGTGCAAAGCTTTTCCAAGATTTTCAGATGT
TAAGTAGAATATGGACTCATCCTTGGTGTTTGCAGCTAGACTACATTAGCAAAGAAAATAAGGGTTATTTTGA
TGAAGACAGTATGGATGAATTTATAGCCTCAGATTCTGATGAAACCTCCATGAGTTTAAGCTCCGATGATTAT
ACAAAAAAGAAGAAAAAAGGGAAAAAGGGGAAAAAAGATAGTAGCTCAAGTGGAAGTGGCAGTGACAATGATG
TTGAAGTGATTAAGGTCTGGAATTCAAGATCTCGGGGAGGTGGTGAAGGAAATGTGGATGAAACAGGAAACAA
TCCTTCTGTTTCTTTAAAACTGGAAGAAAGTAAAGCTACTTCTTCTTCTAATCCAAGCAGCCCAGCTCCAGAC
TGGTACAAAGATTTTGTTACAGATGCTGATGCTGAGGTTTTAGAGCATTCTGGGAAAATGGTACTTCTCTTTG
AAATTCTTCGAATGGCAGAGGAAATTGGGGATAAAGTCCTTGTTTTCAGCCAGTCCCTCATATCTCTGGACTT
GATTGAAGATTTTCTTGAATTAGCTAGTAGGGAGAAGACAGAAGATAAAGATAAACCCCTTATTTATAAAGGT
GAGGGGAAGTGGCTTCGAAACATTGACTATTACCGTTTAGATGGTTCCACTACTGCACAGTCAAGGAAGAAGT
GGGCTGAAGAATTTAATGATGAAACTAATGTGAGAGGACGATTATTTATCATTTCTACTAAAGCAGGATCTCT
AGGAATTAATCTGGTAGCTGCTAATCGAGTAATTATATTCGACGCTTCTTGGAATCCATCTTATGACATCCAG
AGTATATTCAGAGTTTATCGCTTTGGACAAACTAAGCCTGTTTATGTATATAGGTTCTTAGCTCAGGGAACCA
TGGAAGATAAGATTTATGATCGGCAAGTAACTAAGCAGTCACTGTCTTTTCGAGTTGTTGATCAGCAGCAGGT
GGAGCGTCATTTTACTATGAATGAGCTTACTGAACTTTATACTTTTGAGCCAGACTTATTAGATGACCCTAAT
TCAGAAAAGAAGAAGAAGAGGGATACTCCCATGCTGCCAAAGGATACCATACTTGCAGAGCTCCTTCAGATAC
ATAAAGAACACATTGTAGGATACCATGAACATGATTCTCTTTTGGACCACAAAGAAGAAGAAGAGTTGACTGA
AGAAGAAAGAAAAGCAGCTTGGGCTGAGTATGAAGCAGAGAAGAAGGGACTGACCATGCGTTTCAACATACCA
ACTGGGACCAATTTACCCCCTGTCAGTTTCAACTCTCAAACTCCTTATATTCCTTTCAATTTGGGAGCCCTGT
CAGCAATGAGTAATCAACAGCTGGAGGACCTCATTAATCAAGGAAGAGAAAAAGTTGTAGAAGCAACAAACAG
TGTGACAGCAGTGAGGATTCAACCTCTTGAGGATATAATTTCAGCTGTATGGAAGGAGAACATGAATCTCTCA
GAGGCCCAAGTACAGGCGTTAGCATTAAGTAGACAAGCCAGCCAGGAGCTTGATGTTAAACGAAGAGAAGCAA
TCTACAATGATGTATTGACAAAACAACAGATGTTAATCAGCTGTGTTCAGCGAATACTTATGAACAGAAGGCT
CCAGCAGCAGTACAATCAGCAGCAACAGCAACAAATGACTTATCAACAAGCAACACTGGGTCACCTCATGATG
CCAAAGCCCCCAAATTTGATCATGAATCCTTCTAACTACCAGCAGATTGATATGAGAGGAATGTATCAGCCAG
TGGCTGGTGGTATGCAGCCACCACCATTACAGCGTGCACCACCCCCAATGAGAAGCAAAAATCCAGGACCTTC
CCAAGGGAAATCAATTGCATACCCATACGATGTTCCAGATTACGCTTGATCTAGAGCGGCCGCCTGCAGCTGG
CGCCATCGATACGCGTACGTCGCGACCGCGGACATGTACAGAGCTCGAGCTCAAGCTTCGAATTCTGCAGTCG
ACGGTACCGCGGGCCCGGGATCCACCGGATCTAGATAACTGATCATAATCAGCCATACCACATTTGTAGAGGT
TTTACTTGCTTTAAAAAACCTCCCACACCTCCCCCTGAACCTGAAACATAAAATGAATGCAATTGTTGTTGTT
AACTTGTTTATTGCAGCTTATAATGGTTACAAATAAAGCAATAGCATCACAAATTTCACAAATAAAGCATTTT
TTTCACTGCATTCTAGTTGTGGTTTGTCCAAACTCATCAATGTATCTTAACGCGTAAATTGTAAGCGTTAATA
TTTTGTTAAAATTCGCGTTAAATTTTTGTTAAATCAGCTCATTTTTTAACCAATAGGCCGAAATCGGCAAAAT
CCCTTATAAATCAAAAGAATAGACCGAGATAGGGTTGAGTGTTGTTCCAGTTTGGAACAAGAGTCCACTATTA
AAGAACGTGGACTCCAACGTCAAAGGGCGAAAAACCGTCTATCAGGGCGATGGCCCACTACGTGAACCATCAC
CCTAATCAAGTTTTTTGGGGTCGAGGTGCCGTAAAGCACTAAATCGGAACCCTAAAGGGAGCCCCCGATTTAG
AGCTTGACGGGGAAAGCCGGCGAACGTGGCGAGAAAGGAAGGGAAGAAAGCGAAAGGAGCGGGCGCTAGGGCG
CTGGCAAGTGTAGCGGTCACGCTGCGCGTAACCACCACACCCGCCGCGCTTAATGCGCCGCTACAGGGCGCGT
CAGGTGGCACTTTTCGGGGAAATGTGCGCGGAACCCCTATTTGTTTATTTTTCTAAATACATTCAAATATGTA
TCCGCTCATGAGACAATAACCCTGATAAATGCTTCAATAATATTGAAAAAGGAAGAGTCCTGAGGCGGAAAGA
ACCAGCTGTGGAATGTGTGTCAGTTAGGGTGTGGAAAGTCCCCAGGCTCCCCAGCAGGCAGAAGTATGCAAAG
CATGCATCTCAATTAGTCAGCAACCAGGTGTGGAAAGTCCCCAGGCTCCCCAGCAGGCAGAAGTATGCAAAGC
ATGCATCTCAATTAGTCAGCAACCATAGTCCCGCCCCTAACTCCGCCCATCCCGCCCCTAACTCCGCCCAGTT
CCGCCCATTCTCCGCCCCATGGCTGACTAATTTTTTTTATTTATGCAGAGGCCGAGGCCGCCTCGGCCTCTGA
GCTATTCCAGAAGTAGTGAGGAGGCTTTTTTGGAGGCCTAGGCTTTTGCAAAGATCGATCAAGAGACAGGATG
AGGATCGTTTCGCATGATTGAACAAGATGGATTGCACGCAGGTTCTCCGGCCGCTTGGGTGGAGAGGCTATTC
GGCTATGACTGGGCACAACAGACAATCGGCTGCTCTGATGCCGCCGTGTTCCGGCTGTCAGCGCAGGGGCGCC
CGGTTCTTTTTGTCAAGACCGACCTGTCCGGTGCCCTGAATGAACTGCAAGACGAGGCAGCGCGGCTATCGTG
GCTGGCCACGACGGGCGTTCCTTGCGCAGCTGTGCTCGACGTTGTCACTGAAGCGGGAAGGGACTGGCTGCTA
TTGGGCGAAGTGCCGGGGCAGGATCTCCTGTCATCTCACCTTGCTCCTGCCGAGAAAGTATCCATCATGGCTG
ATGCAATGCGGCGGCTGCATACGCTTGATCCGGCTACCTGCCCATTCGACCACCAAGCGAAACATCGCATCGA
GCGAGCACGTACTCGGATGGAAGCCGGTCTTGTCGATCAGGATGATCTGGACGAAGAGCATCAGGGGCTCGCG
CCAGCCGAACTGTTCGCCAGGCTCAAGGCGAGCATGCCCGACGGCGAGGATCTCGTCGTGACCCATGGCGATG
CCTGCTTGCCGAATATCATGGTGGAAAATGGCCGCTTTTCTGGATTCATCGACTGTGGCCGGCTGGGTGTGGC
GGACCGCTATCAGGACATAGCGTTGGCTACCCGTGATATTGCTGAAGAGCTTGGCGGCGAATGGGCTGACCGC
TTCCTCGTGCTTTACGGTATCGCCGCTCCCGATTCGCAGCGCATCGCCTTCTATCGCCTTCTTGACGAGTTCT
TCTGAGCGGGACTCTGGGGTTCGAAATGACCGACCAAGCGACGCCCAACCTGCCATCACGAGATTTCGATTCC

ACCGCCGCCTTCTATGAAAGGTTGGGCTTCGGAATCGTTTTCCGGGACGCCGGCTGGATGATCCTCCAGCGCG
GGGATCTCATGCTGGAGTTCTTCGCCCACCCTAGGGGGAGGCTAACTGAAACACGGAAGGAGACAATACCGGA
AGGAACCCGCGCTATGACGGCAATAAAAAGACAGAATAAAACGCACGGTGTTGGGTCGTTTGTTCATAAACGC
GGGGTTCGGTCCCAGGGCTGGCACTCTGTCGATACCCCACCGAGACCCCATTGGGGCCAATACGCCCGCGTTT
CTTCCTTTTCCCCACCCCACCCCCCAAGTTCGGGTGAAGGCCCAGGGCTCGCAGCCAACGTCGGGGCGGCAGG
CCCTGCCATAGCCTCAGGTTACTCATATATACTTTAGATTGATTTAAAACTTCATTTTTAATTTAAAAGGATC
TAGGTGAAGATCCTTTTTGATAATCTCATGACCAAAATCCCTTAACGTGAGTTTTCGTTCCACTGAGCGTCAG
ACCCCGTAGAAAAGATCAAAGGATCTTCTTGAGATCCTTTTTTTCTGCGCGTAATCTGCTGCTTGCAAACAAA
AAAACCACCGCTACCAGCGGTGGTTTGTTTGCCGGATCAAGAGCTACCAACTCTTTTTCCGAAGGTAACTGGC
TTCAGCAGAGCGCAGATACCAAATACTGTCCTTCTAGTGTAGCCGTAGTTAGGCCACCACTTCAAGAACTCTG
TAGCACCGCCTACATACCTCGCTCTGCTAATCCTGTTACCAGTGGCTGCTGCCAGTGGCGATAAGTCGTGTCT
TACCGGGTTGGACTCAAGACGATAGTTACCGGATAAGGCGCAGCGGTCGGGCTGAACGGGGGGTTCGTGCACA
CAGCCCAGCTTGGAGCGAACGACCTACACCGAACTGAGATACCTACAGCGTGAGCTATGAGAAAGCGCCACGC
TTCCCGAAGGGAGAAAGGCGGACAGGTATCCGGTAAGCGGCAGGGTCGGAACAGGAGAGCGCACGAGGGAGCT
TCCAGGGGGAAACGCCTGGTATCTTTATAGTCCTGTCGGGTTTCGCCACCTCTGACTTGAGCGTCGATTTTTG
TGATGCTCGTCAGGGGGGCGGAGCCTATGGAAAAACGCCAGCAACGCGGCCTTTTTACGGTTCCTGGCCTTTT
GCTGGCCTTTTGCTCACATGTTCTTTCCTGCGTTATCCCCTGATTCTGTGGATAACCGTATTACCGCCATGCA
T

>Primers for ATRX PCR analysis

Ax I – F	ATGACCGCTGAGCCCATG
Ax I – R	GGAAGGAACAGACACAATATCCATG
Ax II – F	GAGCACATGCATCAGAATGTTCC
Ax II – R	GGAATATTTTCTCAGTATCAGATGATGAACAATC
Ax III – F	GCTGAGAAGTCAACAGGGAAAG
Ax III – R	GCAATCCCACATAAACTGAACAC
Ax IV – F	GCAAAGAAAGCAGAGTTGGAAGA
Ax IV – R	GGAACCATCTAAACGGTAATAGTCAATG
Ax V – F	GGAAGTGGCAGTGACAATGATG
Ax V – R	CATTGATTTCCCTTGGGAAGGTC

>Exon8-F primer (sequencing)
GCCAACATGAACTCCAGTTATG

>ATRX-IS10_insertion_site-F primer (colony PCR)
GGCTTTTAAGTCTGTGTTGGC
>ATRX- IS10_insertion_site-R primer (colony PCR)
CCATAGCAGTCTCAAGATTCTCAA
